# Predictive model for neonatal HBV infection risk in infants of HBV-infected mothers in China: an observational study

**DOI:** 10.3389/fpubh.2025.1536904

**Published:** 2025-04-03

**Authors:** Xingzhu Liu, Chuxiong Gong, Xiaoliang Du, Yanfei Yang, Liyue Kui, Lin Wang, Tingting Hao, Yao Hou, Feng Wang, Na Fan, Yuqin Wu

**Affiliations:** ^1^Department of Special Needs Ward, Kunming Children’s Hospital, Kunming, China; ^2^Department of Cardiovascular Medicine, Kunming Children’s Hospital, Kunming, China; ^3^Department of Science and Education, Kunming Children’s Hospital, Kunming, China; ^4^Department of Laboratory, Kunming Children’s Hospital, Kunming, China; ^5^Institute of Medical Biology, Chinese Academy of Medical Sciences, Kunming, China

**Keywords:** vertical transmission, hepatitis B virus, predictive model, direct bilirubin, gamma-glutamyl transferase, HBsAg, HBcAb

## Abstract

**Objective:**

In China, vertical transmission is the primary route of hepatitis B virus (HBV) transmission from mothers to their children. This study aimed to develop a predictive model for assessing the risk of HBV infection in newborns born to mothers with HBV infection. Additionally, the model was validated across subgroups based on child stage, gender, and race to facilitate the early identification of high-risk newborns and the development of personalized preventive measures.

**Methods:**

We collected medical records of 443 newborns whose mothers had a history of hepatitis B. We compared case characteristics between newborns with and without HBV infection and identified key factors using LASSO approach to construct a multivariate logistic regression prediction model. The model’s performance was evaluated using the ROC curve, calibration curve, and decision curve analysis. The stability of the predictions was further validated through 5-fold cross-validation. Finally, subgroup analyses were conducted based on sex, age, and race.

**Results:**

We identified alanine aminotransferase, direct bilirubin, gamma-glutamyl transferase, HBsAg, and HBcAb as key factors for the prediction model. The model achieved an area under the ROC curve of 0.890 (95% CI: 0.831–0.949). The calibration curve and decision curve analysis confirmed the model’s accuracy, and the 5-fold cross-validation reaffirmed its internal stability. The model also demonstrated robust validation across different age, gender, and race subgroups.

**Conclusion:**

Our study developed a reliable predictive model for assessing the risk of HBV infection among newborns of HBV-infected mothers in China. The model performed well across various child stages, genders, and racial subgroups. This research provides a foundation for the early identification of newborns at high risk for HBV infection, thereby reducing the risk of neonatal HBV transmission and supporting the rationale for individualized precision treatment.

## 1 Introduction

Hepatitis B is a potentially life-threatening infectious disease caused by the HBV. This infection poses a significant public health challenge worldwide. Over 2 billion people have been exposed to HBV, with an estimated 387 million currently living with chronic infections and approximately 10 million new carriers emerging each year. China has one of the highest rates of hepatitis B globally, and the incidence continues to rise (1, 2). The primary transmission routes of hepatitis B include blood transmission and mother-to-child transmission.

In China, chronic hepatitis B infection is typically transmitted to newborns by mothers who are chronic carriers during delivery. Mothers who are positive for hepatitis B e antigen (HBeAg) or have a high maternal HBV DNA load usually present with elevated viral loads and increased infectiousness (3). The risk of vertical transmission is most pronounced in mothers who test positive for both HBsAg and HBeAg, with transmission rates estimated between 70 and 90%. In contrast, for mothers who are HBsAg positive but HBeAg negative, the risk decreases from 40% to around 10% (4, 5). Studies indicate that the risk of perinatal HBV infection in infants of HBsAg-positive mothers varies widely, ranging from less than 10% to as high as 70 to 90%, depending on maternal HBeAg status (6, 7). Additionally, the degree of liver damage or the liver’s compensatory ability before delivery plays a crucial role in determining the risk of HBV transmission to infants. Mothers with impaired liver function are more likely to transmit the virus to their newborns (8). Factors such as maternal gestational age and overall health also influence transmission risk. Passive-active combined immunization can significantly reduce the mother-to-child transmission rate during the neonatal period, decreasing it from 75 to 90% to around 10%. However, there remains some debate regarding the relationship between maternal infection and the neonatal immune response (9).

Although existing immunological prophylaxis has significantly reduced the risk of transmission, early identification of high-risk cases can further mitigate the risk of HBV infection. Reducing maternal viral load during pregnancy and increasing the HBV vaccine dose for infants are critical strategies. Our study aimed to establish a predictive model for hepatitis B infection in newborns born to mothers with a history of hepatitis B. By identifying newborns at high risk for HBV infection early, we can develop personalized preventive measures to further reduce the risk of infection in this vulnerable population.

## 2 Materials and methods

### 2.1 Participants

Our study retrospectively collected cases and relevant clinical data of hospitalized newborns at Kunming Children’s Hospital from January 2020 to January 2024. The mothers of these newborns had a history of hepatitis B infection. All mothers underwent hepatitis B antigen and antibody testing before pregnancy, including HBsAg, HBsAb, HBeAg, HBeAb, and HBcAb, with all tests conducted using enzyme-linked immunosorbent assay (ELISA). All mothers with a history of HBV infection received treatment at hospitals with extensive experience in HBV management. None of the mothers had received hepatitis B vaccinations within three years before pregnancy. The presence of hepatitis B infection in all included newborns was confirmed by HBV DNA testing, with newborns having HBV DNA >20 IU/mL considered to be infected with HBV. All included newborns received basic immunoprophylaxis with hepatitis B vaccine and hepatitis B immunoglobulin (HBIG) within 12 h of birth. Informed consent was obtained from the parents of all participants for the use of case data and maternal medical history.

### 2.2 Inclusion and exclusion criteria

Inclusion criteria: (1) The mother had a confirmed diagnosis of HBV infection; (2) Complete clinical data for both the newborn and the mother. Exclusion criteria: (1) The newborn had underlying diseases such as liver disease, kidney disease, hematological disorders, immune system diseases, or hereditary metabolic endocrine disorders; (2) The mother had not undergone professional assessment and regular treatment for hepatitis B during her infection; (3) The mother had a history of hepatitis B exposure during pregnancy; (4) The mother had a history of blood transfusion during pregnancy; (5) The newborn did not receive HBIG treatment within 12 h; (6) The newborn had a history of hepatitis B exposure prior to HBV DNA testing.

### 2.3 Included factors

Eighteen relevant factors were included in the analysis. These encompassed the child’s basic demographic information (such as age, sex, ethnicity); maternal serological test results for hepatitis B prior to pregnancy (HBsAg, HBsAb, HBeAg, HBeAb, HBcAb); routine blood and biochemical indicators [alanine aminotransferase (ALT), aspartate aminotransferase (AST), gamma-glutamyl transferase (GGT), direct bilirubin (DBIL), albumin, hepatitis B virus (HBV), immunoglobulin M (IgM), immunoglobulin G (IgG), and alkaline phosphatase (ALP)]. Additionally, maternal medical history information was included, such as gestational age, alcohol consumption history, blood transfusion history, and family history.

### 2.4 Model construction

All newborn cases were first divided into HBV and non-HBV groups based on HBV infection status. We compared the eighteen relevant factors between the two groups. Then, the statistically significant factors were included in the subsequent analysis. We then used the “glmnet” R package to perform Least Absolute Shrinkage and Selection Operator (LASSO) for further selection of the factors that are more important for predicting the outcome. LASSO is a regularization method for linear regression. It achieves variable selection and model simplification by introducing an L1 regularization term into the loss function. The main advantages of LASSO include its ability to shrink the coefficients of unimportant variables to zero, thereby retaining only the key variables, reducing model complexity to prevent overfitting. During the selection process, 5-fold cross-validation was used to select the optimal lambda value, namely lambda.min or lambda.1se. A larger lambda indicates a stronger regularization effect of the model, resulting in fewer selected independent variables. Variables were selected based on the optimal and maximum lambda values. Then, we used the “rmda” R package to include the finally selected factors in the multivariable logistic regression analysis to construct the prediction model. We also used the “rms” R package to plot the nomogram of the optimal model.

### 2.5 Model evaluation and validation

We first utilized the “pROC” R package to plot the receiver operating characteristic (ROC) curve and assess model performance using the area under the curve (AUC). Next, we employed the “ResourceSelection” R package to create a calibration curve to evaluate the model’s calibration, followed by decision curve analysis (DCA) using the “decision_curve” function for further accuracy assessment. Finally, we conducted 5-fold cross-validation using the createFolds function.

### 2.6 Subgroup analysis

We categorized all cases by child sex into male and female; by age into 1 week, 2 weeks, 3 weeks, and 4 weeks; and by ethnicity into Ethnic minorities and Han ethnicity. We then calculated the AUC for each subgroup to evaluate the model’s predictive value.

### 2.7 Statistical analysis

All statistical analyses were performed using R version 4.4.1. For normally distributed continuous data, results were expressed as mean ± SD, and independent samples t-tests were used for group comparisons. For non-normally distributed data, quartiles were used for description, and non-parametric rank-sum tests were employed for comparisons. Categorical data were expressed as proportions, with chi-square tests used for comparisons between groups. A *p*-value of <0.05 was considered statistically significant.

## 3 Results

### 3.1 Description and comparison of clinical characteristics

Among the mothers included in the study, 15 cases were diagnosed with chronic active hepatitis B based on pre-pregnancy hepatitis B antigen and antibody testing results, of which 8 newborns were infected with HBV; 7 cases were diagnosed with chronic inactive hepatitis B, of which 4 newborns were infected with HBV; and there were 421 cases of mothers with a past hepatitis B infection, among which 33 newborns were infected with HBV. The proportion of HBV infection in newborns born to mothers with a past hepatitis B infection was significantly lower than that in the other two groups (*p* < 0.05).

Among the included patients, 45 (10.16%) were in the HBV group, while 398(89.84%) were in the n-HBV group. A comparison of clinical data revealed significant differences in ALT, AST, DBIL, GGT, HBeAg, HBsAg, HBeAb, and HBcAb between the two groups (Table 1).

**Table 1:** Comparison of clinical data between the HBV and n-HBV groups.

Factor	HBV (*n*=45)	n-HBV (*n*=398)	*p*
Newborn’s age (day)	10.76±9.89	9.05±7.15	0.266
Newborn’s sex			0.999
Female	19(42.22)	166(41.71)	
Male	26(57.78)	232(58.29)	
Newborn’s ethnicity			0.355
Han ethnicity	39(86.67)	317(79.65)	
Ethnic minorities	6(13.33)	81(20.35)	
ALT	58.13±24.78	39.66±15.56	<0.001
AST	53.78±27.4	39.39±15.46	0.001
DBIL	25.29±8.32	19±5.55	<0.001
Albumin	34.52±3.86	34.83±15.76	0.75
IgM	2.34±0.71	2.31±0.58	0.805
ALP	479.62±215.74	433.97±154.53	0.174
GGT	25(18-30)	16(10-19)	<0.001
HB	110(102-116)	112(102-119)	0.984
IgG	15(11.44-19)	14(10.12-17)	0.177
Gestational age in days	269(264-273)	268(264-274)	0.790
HBsAg			<0.001
No	33(73.33)	388(97.49)	
Yes	12(26.67)	10(2.51)	
HBsAb			0.638
No	22(48.89)	175(43.97)	
Yes	23(51.11)	223(56.03)	
HBeAg			0.001
No	33(73.33)	386(96.98)	
Yes	12(26.67)	12(3.02)	
HBeAb			<0.001
No	25(55.56)	313(78.64)	
Yes	20(44.44)	85(21.36)	
HBcAb			<0.001
No	15(33.33)	275(69.1)	
Yes	30(66.67)	123(30.9)	
Alcohol history			0.110
No	27(60)	289(72.61)	
Yes	18(40)	109(27.39)	
Family history			0.999
No	27(60)	241(60.55)	
Yes	18(40)	157(39.45)	
Blood transfusion history			0.463
No	35(77.78)	284(71.36)	
Yes	10(22.22)	114(28.64)	

### 3.2 Screening of key factors

**Figure 1:**
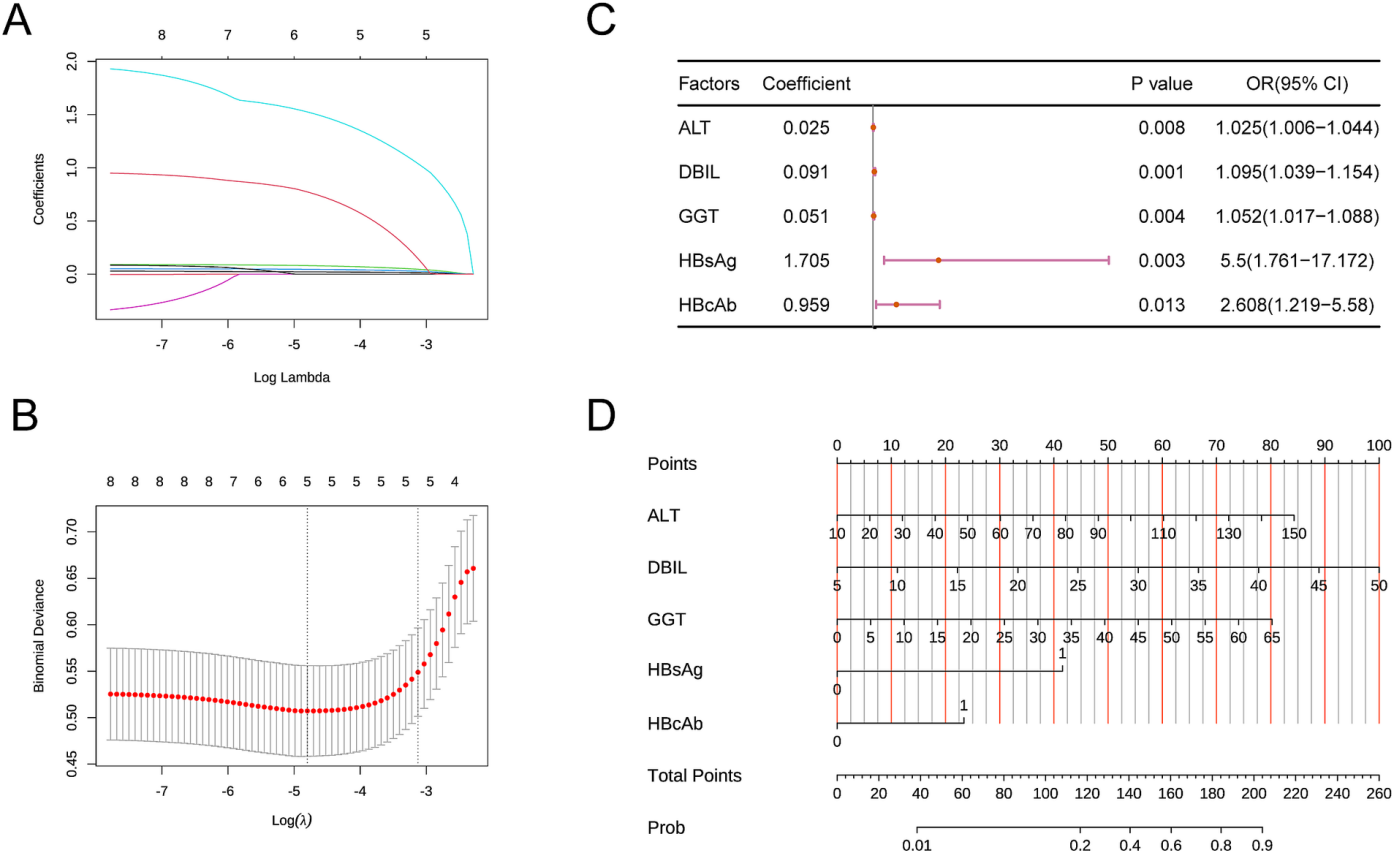
**(A,B)** Key variables selected by LASSO. **(C)** Forest plot of the multivariable logistic regression model. **(D)** Nomogram of the predictive model. Direct Bilirubin (DBIL), Gamma-Glutamyl Transferase (GGT), Hepatitis B Surface Antigen (HBsAg), Hepatitis B e Antigen (HBeAg), Aspartate Aminotransferase (AST), Alanine Aminotransferase (ALT), Hepatitis B Surface Antibody (HBsAb), Hepatitis B Core Antibody (HBcAb).

We included the eight factors mentioned above in the LASSO analysis to select the factors with greater predictive value. During the selection process, 5-fold cross-validation was used to choose the optimal lambda value, which was found to be 0.0082. The factors ALT, DBIL, GGT, HBAg, and HBcAb were identified as having the best linear relationships (Figures 1A,B) and were selected as the final key variables for the subsequent construction of the multivariable logistic model.

### 3.3 Construction and evaluation of the prediction model

**Figure 2:**
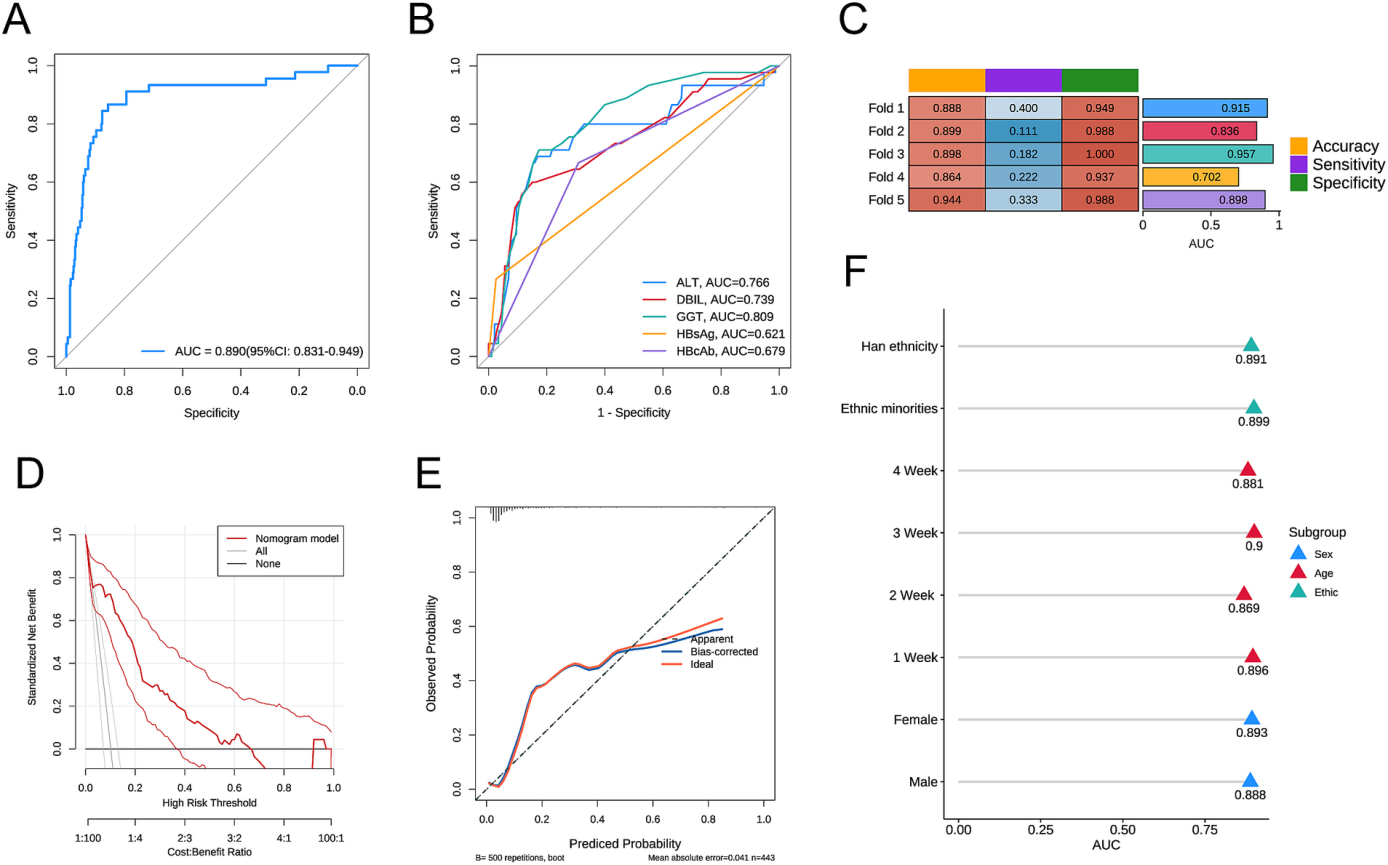
**(A)** ROC curve of the predictive model; **(B)** ROC curves for individual factors DBIL, GGT, HBeAg, and HBsAg; **(C)** Results of the 5-fold cross-validation; **(D)** Calibration curve of the predictive model; **(E)** DCA curve of the predictive model; **(F)** AUC values for each subgroup. Direct Bilirubin (DBIL), Gamma-Glutamyl Transferase (GGT), Hepatitis B Surface Antigen (HBsAg), Hepatitis B e Antigen (HBeAg).

First, we included the AUC in the multivariable logistic regression model. Higher levels of ALT, higher levels of DBIL, higher levels of GGT, HBsAg positivity, and HBcAb positivity were identified as statistically significant independent risk factors for the outcome. A forest plot was used to display the analysis results (Figure 1C). A nomogram was created to visualize the prediction model (Figure 1D). The model’s AUC was 0.890 (95% CI: 0.831–0.949) (Figure 2A). The model’s AUC was significantly greater than the AUC of individual factors (Figure 2B). We also performed 5-fold cross-validation on the model. This result indicates that our constructed model has good predictive performance (Figure 2C). Finally, the calibration curve and DCA curve further confirmed that the model has accurate and valuable predictive significance (Figures 2D,E).

### 3.4 Subgroup analysis

To assess the predictive efficacy of our model under various conditions, we performed a subgroup analysis based on sex, age, and ethnicity. In each subgroup, the model demonstrated good predictive value (Figure 2F). Among them, the AUC of the 3-week subgroup was the highest at 0.900, while the AUC of the 2-week subgroup was the lowest at 0.869.

## 4 Discussion

China has a high incidence of HBV infection, with mother-to-child transmission being a significant contributor. If no measures are taken, the risk of vertical transmission can reach 90% (10). Timely prediction of vertical transmission enables early prevention of neonatal hepatitis B infection (11, 12). In this study, we analyzed medical records from 443 newborns with a maternal history of hepatitis B. We compared the characteristics of newborns with and without HBV infection, identifying DBIL, GGT, HBsAg, and HBeAg as key predictive factors. Our multivariate logistic regression model demonstrated strong predictive power, validated through 50% cross-validation. Analyses based on sex, age, and ethnicity confirmed the model’s effectiveness across subgroups.

HBV is a widely spread infectious disease globally, and vertical transmission or early-life acquisition of the infection is a leading cause of chronic hepatitis, cirrhosis, and even hepatocellular carcinoma (HCC) (13–16). Studies showed that approximately 90% of newborns and infants infected with HBV developed chronic infections, while the chronicity rate for adults was only 5% to 10% (17). This high chronicity rate closely related to the underdeveloped immune system in infants, which is unable to effectively clear the virus. Chronic HBV infection not only leads to persistent liver damage but also may further progress to cirrhosis and HCC, posing a serious threat to patients’ health (18, 19). Therefore, preventing neonatal HBV infection is a critical step in blocking the occurrence of chronic liver disease and liver cancer. Preventing chronic liver disease and liver cancer by addressing neonatal HBV infection holds significant public health implications. First, the high chronicity rate of HBV infection in newborns often goes unnoticed as chronic infections are usually asymptomatic, becoming evident only when they progress to cirrhosis or HCC, which complicates treatment and worsens prognosis (20). Second, chronic HBV infection is a long-term process, potentially taking decades to progress from infection to cirrhosis or liver cancer; once it enters the chronic phase, the virus becomes difficult to eradicate, necessitating lifelong monitoring and treatment, thus imposing heavy economic and psychological burdens on families and society (21). Therefore, blocking vertical transmission of HBV at its source not only significantly reduces the incidence of chronic liver disease and liver cancer but also alleviates the long-term impact of the disease on individuals and society. To effectively prevent neonatal HBV infection, multi-layered comprehensive intervention measures are required. First, all pregnant women should undergo HBV screening during pregnancy (including HBsAg, HBeAg, and HBV-DNA testing), and antiviral treatment should be initiated in the late pregnancy period for those with high viral loads (HBV-DNA ≥10^6 IU/mL) to reduce the risk of mother-to-child transmission (22). Second, during delivery, strict measures should be taken to avoid neonatal contact with maternal blood or body fluids, and the appropriate delivery method should be selected based on obstetric indications. Most importantly, high-titer hepatitis B immunoglobulin (HBIG) should be administered within 12 h of birth, followed by the first dose of hepatitis B vaccine within 24 h; these are core measures to block mother-to-child transmission (23). For newborns of mothers with high viral loads, it is recommended to administer an additional 200 U of HBIG at 14 days and ensure the completion of the hepatitis B vaccine schedule (0, 1, and 6 months). Furthermore, breastfeeding is generally safe after immunization, but care should be taken to avoid breastfeeding when there are cracks or bleeding from the nipples.

Long-term follow-up and regular assessments are essential for ensuring the effectiveness of prevention strategies. Newborns should undergo HBsAg and anti-HBs testing between 9 and 12 months of age to evaluate the effects of immune prevention and infection status. For uninfected newborns, regular monitoring of anti-HBs levels should take place, with booster vaccinations as necessary; for infected children, liver function, HBV-DNA levels, and liver imaging changes should be regularly evaluated for early detection and treatment of chronic liver disease or liver cancer (24). Breastfeeding is usually safe after immunization, but should avoid breastfeeding during nipple breakage or bleeding. Through health education, optimized allocation of public health resources, and multidisciplinary collaboration, vertical transmission can be more precisely interrupted, reducing the risks of chronic hepatitis and liver cancer, thus providing a healthier future for newborns.

HBcAb is an antibody against the HBV core antigen and usually appears early in HBV infection, reflecting the immune response of the body to the virus. In the context of mother-to-child transmission, the presence of HBcAb serves as an important predictor of hepatitis B transmission. Studies indicated that HBcAb-positive mothers tended to have higher HBV viral loads during pregnancy, increasing the risk of the virus being transmitted to the newborn through delivery or breast milk. Additionally, the appearance of HBcAb may suggest active viral replication in the mother, further influencing the likelihood of mother-to-child transmission. HBsAg (hepatitis B surface antigen) is a marker secreted into circulation during HBV replication. Maternal exposure to HBsAg may induce partial immune tolerance in the fetus, weakening the immune response of the newborn to the hepatitis B vaccine and leading to vaccination failure (23). Among the multiple factors influencing vertical transmission, maternal HBV-DNA levels positively correlated with viral DNA in umbilical cord blood, emphasizing the importance of antiviral therapy during pregnancy (24). Furthermore, HBV sequestration within placental villous capillaries may increase the risk of transplacental transmission, especially when viral concentrations are high on the placental surface (25). The presence of maternal HBsAg may also induce fetal immune tolerance during early pregnancy, resulting in inadequate immune responses post-vaccination (26). Liver function closely correlates with hepatitis B transmission; impaired liver function often accompanies active HBV replication and high HBV-DNA levels, thereby increasing the risk of transmission. Liver insufficiency may suppress the immune system, further exacerbating the likelihood of transmission (27). Additionally, patients with abnormal liver function may require adjustments to antiviral drug dosages, which may affect viral clearance and transmission risk (28). In general, good liver function helps control viral replication and reduces transmission risk, whereas abnormal liver function may worsen it. The maternal HBV infection status and liver function indicators directly influence the risk of neonatal infection. Pregnant women who are HBV carriers with abnormal liver function and high viral loads exhibited a significantly increased risk of neonatal infection (29); conversely, those with normal liver function and low viral activity had a lower risk. Timely preventive measures, such as administering immunoglobulin and vaccination at delivery, effectively reduced vertical transmission risks (30, 31). Therefore, monitoring and managing maternal HBV infection and liver function is crucial for neonatal health.

Our study identified elevated ALT, DBIL, and GGT as important indicators for predicting neonatal HBV infection. In clinical practice, this model can serve as an optimal diagnostic tool for identifying pediatric HBV infection. First, routine testing of HBsAg and HBcAb should occur during antenatal screening to assess HBV infection status. Second, combining liver function indicators such as ALT, DBIL, and GGT can help evaluate the activity of viral replication and liver health. Finally, using the model to calculate a risk score allows for the identification of high-risk newborns. For high-risk infants, immediate intervention measures, such as administering hepatitis B immunoglobulin (HBIG) and vaccination, may be taken after birth to interrupt mother-to-child transmission. Additionally, the model can guide antiviral therapy during pregnancy by reducing maternal viral loads and further decreasing transmission risks. Through this stratified management and precise intervention strategy, we can significantly reduce the incidence of pediatric HBV infection and improve outcomes for affected children.

As a retrospective study, our research carries some inherent bias. Additionally, the subjects lacked external validation across different regions, ethnicities, and time periods. To enhance the reliability of our findings and reduce bias, future studies should prioritize large-scale, multicenter, prospective designs that include diverse populations.

## 5 Conclusion

In conclusion, our study constructs a predictive model with strong efficacy for assessing the risk of HBV infection in newborns born to HBV-infected mothers in regional China. The model also demonstrates good validation across subgroups based on child age, sex, and ethnicity. Our findings provide a foundation for early identification of newborns at risk for HBV infection, enabling the development of personalized preventive measures to further reduce infection risk and support individualized treatment approaches.

## Data Availability

The original contributions presented in the study are included in the article/supplementary material, further inquiries can be directed to the corresponding author/s.
